# Impact of perioperative RSV or influenza infection on length of stay and risk of unplanned ICU admission in children: a case-control study

**DOI:** 10.1186/1471-2253-11-16

**Published:** 2011-09-05

**Authors:** Michael C Spaeder, Justin L Lockman, Robert S Greenberg, James C Fackler, Joanne Shay

**Affiliations:** 1 Department of Pediatrics, The George Washington University School of Medicine and Health Sciences and the Division of Critical Care Medicine, Children's National Medical Center, Washington, DC. USA; 2 Division of Pediatric Anesthesia and Critical Care Medicine, The Johns Hopkins Medical Institutions, Baltimore, MD. USA

## Abstract

**Background:**

Children with viral respiratory infections who undergo general anesthesia are at increased risk of respiratory complications. We investigated the impact of RSV and influenza infection on perioperative outcomes in children undergoing general anesthesia.

**Methods:**

We performed a retrospective case-control study. All patients under the age of 18 years who underwent general anesthesia at our institution with confirmed RSV or influenza infection diagnosed within 24 hours following induction between October 2002 and September 2008 were identified. Controls were randomly selected and were matched by surgical procedure, age, and time of year in a ratio of three controls per case. The primary outcome was postoperative length of stay (LOS).

**Results:**

Twenty-four patients with laboratory-confirmed RSV or influenza who underwent general anesthesia prior to diagnosis of viral infection were identified and matched to 72 controls. Thirteen cases had RSV and 11 had influenza. The median postoperative LOS was three days (intra-quartile range 1 to 8 days) for cases and two days (intra-quartile range 1 to 5 days) for controls. Patients with influenza had a longer postoperative LOS (p < 0.001) and patients with RSV or influenza were at increased risk of unplanned admission to the PICU (p = 0.04) than matched controls.

**Conclusions:**

Our results suggest that children with evidence of influenza infection undergoing general anesthesia, even in the absence of symptoms previously thought to be associated with a high risk of complications, may have a longer postoperative hospital LOS when compared to matched controls. RSV and influenza infection was associated with an increased risk of unplanned PICU admission.

## Background

Screening for symptomatic respiratory illness is a key element of the pediatric preoperative anesthesia assessment. Children with upper respiratory illnesses (URI) who undergo general anesthesia are at increased risk of respiratory complications [1] including laryngospasm [2], bronchospasm and arterial desaturation [3]. Although evidence of systemic illness (i.e. fever, toxic appearance) or lower respiratory tract disease (i.e. productive cough, wheezing) are relative contraindications for elective anesthetics, research suggests that children with uncomplicated URI symptoms can undergo general anesthesia without significant increase in anesthetic complications [4]. Data associating risk of perioperative complications with specific etiologic agents are largely absent from the literature.

Respiratory syncytial virus (RSV) is an enveloped RNA paramyxovirus that causes annual epidemics during winter and early spring in temperate climates [5]. RSV is a ubiquitous virus in the United States with nearly all children undergoing at least one infection by age two and re-infection in adulthood is common [5]. Influenza is an orthomyxovirus, which produces seasonal outbreaks predominately in the winter months in temperate climates [5]. A study by Manworren et al demonstrated that preoperative assessment of patients (history and physical exam) by pediatric anesthesiologists could not predict the presence of active RSV infection [6].

The goal of the present study is to determine whether perioperative outcomes are negatively impacted and the incidence of complications is higher for patients who have occult RSV or influenza virus infection at the time of anesthetic induction than for matched controls.

## Methods

The Institutional Review Board at the Johns Hopkins Hospital reviewed and approved this study. We performed a retrospective case-control study identifying all patients under the age of 18 years who underwent induction of general anesthesia at the Johns Hopkins Hospital between October 2002 and September 2008 and who were subsequently diagnosed with laboratory-confirmed RSV or influenza. Viral infection was defined as identification of RSV or influenza virus by immunochromotography, direct fluorescent antibody, hemeadsorption, tube viral culture or shell vial culture from a nasopharyngeal or endotracheal specimen obtained at the time of, or within 24 hours of, induction of general anesthesia. Patients known to have RSV or influenza virus infection prior to induction were excluded from the study.

Review of the patient chart and clinical databases using a standardized data collection tool was conducted to establish postoperative length of stay, prolonged positive pressure ventilation, need for extracorporeal life support (ECLS), and mortality. The anesthesia record was reviewed to determine pre-anesthesia history including assessment of the presence or absence of fever, symptoms of upper respiratory infections such as cough and rhinorrhea, and passive smoke exposure. We defined perioperative and postoperative events as bronchospasm, laryngospasm or unplanned re-intubation in the operating room or postanesthesia care unit (PACU). Unplanned PICU admission was defined as patients that were preoperatively scheduled for recovery in the PACU but required PICU admission. Prolonged postoperative positive pressure ventilation (PPV) was defined as the requirement of PPV for more than six hours following admission to the PICU. The primary outcome of interest was postoperative length of stay.

Controls were randomly selected from a hospital clinical database for the years 1999 to 2010, and were individually matched with cases by surgical procedure, age, and time of year in a ratio of three controls per case. Previous research at our institution has established the predominant time frame for annual seasonal RSV and influenza epidemics extends from the months of November to March [7]. For the purposes of matching cases and controls on time of year, we designated the months from November to March as the viral season and the months of April to October as the non-viral season.

We identified patients with chronic medical conditions associated with an increased risk of complications from viral illness [8-11]. These conditions included the following: 1) chronic pulmonary conditions such as cystic fibrosis, restrictive lung disease, or chronic lung disease as diagnosed by a pediatric pulmonologist (e.g. chronic supplemental oxygen requirement and/or pharmacological therapies); 2) unrepaired or palliated cyanotic heart disease; 3) gestational age less than 32 weeks and chronological age less than 12 months; 4) pregnancy; 5) immunosuppression as a result of medications following solid organ or bone marrow transplantation, neutropenia or human immunodeficiency virus; 6) neuromuscular disorders such as muscular dystrophy, motor neuron disease (e.g. spinal muscular atrophy) and cerebral palsy; and 7) hemoglobinopathy.

Continuous variables were compared using Wilcoxon matched pairs signed rank testing. Categorical variables were compared using McNemar's testing for matched pairs or McNemar's Exact testing as appropriate. All statistical calculations were performed using Stata/IC 10.1 (Stata Corporation, College Station, TX).

## Results

There were 24 patients with laboratory confirmed RSV or influenza virus infection who underwent general anesthesia prior to diagnosis of viral infection during the period of study. Thirteen patients had RSV and 11 patients had influenza (91% with influenza A). Fifty-four percent of the patients were female and the median age was 10 months (intra-quartile range (IQR) 5 to 28 months). Eighty-three percent of the surgical procedures were conducted during the months of November to March.

A total of 72 controls were randomly selected, matched at a ratio of three controls to each case. Forty-two percent of the controls were female and the median age was 10 months (IQR 6 to 27 months). Baseline characteristics of the cases and their matched controls analyzed by virus are shown in Table 1.

**Table 1:** Preoperative Characteristics of Cases Compared to Controls

Variable	RSV Cases (n = 13) No. (%)	RSV Controls (n = 39) No. (%)	p-value	Influenza Cases (n = 11) No. (%)	Influenza Controls (n = 33) No. (%)	p-value
Age (months)	10 (IQR 5 - 17)	9 (IQR 4 - 19)	NS	22 (IQR 5 - 29)	21 (IQR 9 - 30)	NS

Female sex	8 (62%)	19 (49%)	NS	5 (45%)	13 (39%)	NS

Chronic medical condition(s)	4 (31%)	14 (36%)	NS	5 (45%)	11 (33%)	NS

Pre-op fever	0	2 (5%)	NS	2 (18%)	4 (12%)	NS

Pre-op URI sxs	7 (54%)	1 (3%)	< 0.001	5 (45%)	7 (21%)	NS

Smoke Exposure	1 (8%)	4 (10%)	NS	1 (9%)	1 (3%)	NS

Abbreviations: IQR, intra-quartile range; URI, upper respiratory infection; sxs, symptoms.

The median postoperative length of stay was three days (IQR 1 to 8 days) for the cases and two days (IQR 1 to 5 days) for the controls. Fifty-four percent of cases were admitted to the PICU following surgery compared to 35% of the controls. Of those patients admitted to the PICU following surgery, 31% of cases were unplanned PICU admissions compared to 17% of the controls. No patients in either group required ECLS or died prior to discharge. Outcomes for cases and matched controls analyzed by virus are shown in Table 2.

**Table 2:** Postoperative Outcomes and Complications

Variable	RSV Cases (n = 13) No. (%)	RSV Controls (n = 39) No. (%)	p-value	Influenza Cases (n = 11) No. (%)	Influenza Controls (n = 33) No. (%)	p-value
Postop LOS (days)	2 (IQR 1 - 3)	2 (IQR 1 - 5)	NS	5 (IQR 2 - 11)	2 (IQR 1 - 5)	< 0.001

Peri/Postop events	1 (8%)	3 (8%)	NS	2 (18%)	2 (6%)	NS

Prolonged PPV	1 (8%)	3 (8%)	NS	2 (18%)	2 (6%)	NS

ECLS	0	0	-	0	0	-

Mortality	0	0	-	0	0	-

Abbreviations: LOS, Length of Stay; IQR, intra-quartile range; PICU, Pediatric Intensive Care Unit; PPV, Positive Pressure Ventilation; ECLS, Extracorporeal Life Support

Patients with influenza had longer postoperative lengths of stay (p < 0.001) than matched controls. There was no difference in postoperative lengths of stay between cases and matched controls in patients with RSV. There was an increased risk of unplanned postoperative PICU admission (p = 0.04) in patients with RSV or influenza as compared to matched controls. There was no difference in the incidence of perioperative or postoperative events, or requirement of prolonged postoperative positive pressure ventilation between cases and controls.

## Discussion

Our results suggest that children with occult influenza undergoing general anesthesia have a longer postoperative hospital length of stay as compared to matched controls. Children undergoing general anesthesia with occult RSV or influenza infection have an increased risk of unplanned PICU admission. We observed no difference between cases and matched controls related to the risk of perioperative complications, requirements for prolonged positive pressure ventilation, or mortality.

It is not uncommon for the pediatric anesthesiologist to encounter a child with URI symptoms preoperatively. Such patients may experience increased airway reactivity in the perioperative period often presenting as coughing, bronchospasm, laryngospasm or hypoxemia. This study quantifies the impact of these complications on our young patients with RSV or influenza infections receiving general anesthesia for surgical procedures. Interestingly, our findings are significant despite a lack of URI symptoms in 50% of cases. This supports the concept that even trained pediatric anesthesiologists are unable to accurately detect infection with these agents, and raises further questions regarding the sensitivity of preoperative assessment of symptoms.

The issue of pre-operative screening of children undergoing surgery for viral respiratory illness is complex. In children with signs or symptoms of serious infection (fever, productive cough, toxic appearance), it should be an easy decision for the anesthesiologist or surgeon to postpone surgery if appropriate. It is the children with mild symptoms, or no symptoms at all, that can prove to be difficult.

While rapid viral testing may provide helpful data for these decisions, there are important testing limitations. The current method for rapid screening of viral respiratory antigens at most institutions is immunochromotography. Despite the high specificity (> 90%) that most commercially available immunochromotography tests possess, sensitivity tends to be low (e.g. approximately 60% sensitive for RSV) [12]. Direct fluorescent antibody testing, with a reported sensitivity of 93% and specificity of 97%, is a more reliable method of testing that, despite requiring specialized equipment, may be a practical alternative to immunochromotography [12]. Definitive testing methods such as shell vial culture or polymerase chain reaction often require one to two days to receive results and thus may not be practical for pre-operative screening.

Our study has several limitations. Initiation of viral testing at our institution during the period of study, whether in the operating room, PACU or inpatient ward, was performed on a case-by-case basis by the responsible anesthesiologist, pediatrician or surgeon. It is reasonable to postulate that some patients with viral infection underwent general anesthesia and were not included in our case sample due to a lack of testing. Not all of our controls have documented negative tests. However, because our routine involved both viral testing for symptomatic patients and procedure postponement for patients with systemic or lower respiratory disease, it is unlikely that patients with occult RSV or influenza infection in perioperative period would have been misclassified as controls. More importantly, if such misclassification did occur, it should only serve to decrease the likelihood that the present study would find a significant difference between groups; thus it may be that our results would show an even greater difference between groups if negative testing results were available for all controls.

Our investigation was limited to the types of viruses for which testing was readily available for the time period of study at our institution. Other viral causes of acute respiratory infections in children include the parainfluenza viruses, rhinoviruses, adenovirus, human metapneumovirus and enteroviruses. With polymerase chain reaction testing becoming increasingly available for these viruses, it will be important to assess the impact these viruses have on perioperative outcomes in children undergoing general anesthesia.

We have no information as to the vaccination status of the children in our study. It is reasonable to consider that children with chronic medical conditions were more likely to have received vaccination for seasonal influenza and as such, may be underrepresented in our sample. Our study was comprised of a relatively small number of cases at a single institution. Further research, including a multicenter trial, would be beneficial for confirmation of our outcomes.

## Conclusions

The current study provides a framework for evaluating the symptomatic child, and predicting the escalation in perioperative care associated with concurrent RSV or influenza infection in children who are undergoing elective surgery. For elective cases during the late fall and winter months, we believe it may be reasonable to test symptomatic children preoperatively for the presence of RSV or influenza virus; future studies are required to evaluate the cost-effectiveness of this practice. Our results indicate that children with RSV and influenza virus infections who undergo elective surgical procedures may be at increased risk for postoperative ICU admission and an increased length of hospital stay. These are discrete end points that should be considered in the discussion of risk and benefits associated with proceeding with elective surgery in the child with URI symptoms.

## Competing interests

The authors declare that they have no competing interests.

## Authors' contributions

MS participated in the study design, data collection and analysis, manuscript preparation and coordinated the day-to-day activities of the project. JL participated in the study design, data collection and analysis, manuscript preparation and assisted in the coordination of the day-to-day activities of the project. RG participated in the study design and manuscript preparation. JF participated in the study design and manuscript preparation. JS participated in the study design, data analysis, and manuscript preparation and oversaw the conduct of the study as senior author. All authors read and approved the final manuscript.

## Pre-publication history

The pre-publication history for this paper can be accessed here:

http://www.biomedcentral.com/1471-2253/11/16/prepub
